# Point-of-Care Versus Central Laboratory Measurements of Hemoglobin, Hematocrit, Glucose, Bicarbonate and Electrolytes: A Prospective Observational Study in Critically Ill Patients

**DOI:** 10.1371/journal.pone.0169593

**Published:** 2017-01-10

**Authors:** Jérôme Allardet-Servent, Melissa Lebsir, Christian Dubroca, Martine Fabrigoule, Sylvie Jordana, Thomas Signouret, Matthias Castanier, Guillemette Thomas, Rettinavelou Soundaravelou, Anne Lepidi, Laurence Delapierre, Guillaume Penaranda, Philippe Halfon, Jean-Marie Seghboyan

**Affiliations:** 1 Service de Réanimation, Hôpital Européen Marseille, Marseille, France; 2 Laboratoire Européen, Laboratoire de Biologie Spécialisée Alphabio, Marseille, France; University of New Orleans, UNITED STATES

## Abstract

**Introduction:**

Rapid detection of abnormal biological values using point-of-care (POC) testing allows clinicians to promptly initiate therapy; however, there are concerns regarding the reliability of POC measurements. We investigated the agreement between the latest generation blood gas analyzer and central laboratory measurements of electrolytes, bicarbonate, hemoglobin, hematocrit, and glucose.

**Methods:**

314 paired samples were collected prospectively from 51 critically ill patients. All samples were drawn simultaneously in the morning from an arterial line. BD Vacutainer tubes were analyzed in the central laboratory using Beckman Coulter analyzers (AU 5800 and DxH 800). BD Preset 3 ml heparinized-syringes were analyzed immediately in the ICU using the POC Siemens RAPIDPoint 500 blood gas system. We used CLIA proficiency testing criteria to define acceptable analytical performance and interchangeability.

**Results:**

Biases, limits of agreement (±1.96 SD) and coefficients of correlation were respectively: 1.3 (-2.2 to 4.8 mmol/L, r = 0.936) for sodium; 0.2 (-0.2 to 0.6 mmol/L, r = 0.944) for potassium; -0.9 (-3.7 to 2 mmol/L, r = 0.967) for chloride; 0.8 (-1.9 to 3.4 mmol/L, r = 0.968) for bicarbonate; -11 (-30 to 9 mg/dL, r = 0.972) for glucose; -0.8 (-1.4 to -0.2 g/dL, r = 0.985) for hemoglobin; and -1.1 (-2.9 to 0.7%, r = 0.981) for hematocrit. All differences were below CLIA cut-off values, except for hemoglobin.

**Conclusions:**

Compared to central Laboratory analyzers, the POC Siemens RAPIDPoint 500 blood gas system satisfied the CLIA criteria of interchangeability for all tested parameters, except for hemoglobin. These results are warranted for our own procedures and devices. Bearing these restrictions, we recommend clinicians to initiate an appropriate therapy based on POC testing without awaiting a control measurement.

## Introduction

Critically ill patients are particularly prone to develop severe variations of their electrolytes, bicarbonate, hemoglobin, and glucose concentrations, either at admission or during the course of their stay [1]. These disturbances may be related to the severity of diseases and to the interventions by physicians [2, 3]. Early detection of life-threatening biological variation is required for clinicians to promptly deliver an appropriate therapy.

Although it is common practice to perform daily blood analyze in intensive care units (ICUs), the results are inexorably delayed by a few hours when processed by a central laboratory (Lab) [4]. Blood gas analyzers have been widely introduced into modern ICUs and offer a unique opportunity to determine measurements at the point-of-care (POC), and in a time frame of 2 minutes, not only for blood gases but also many others biological variables [5].

Whether results provided by POC testing are reliable enough remains controversial and some clinicians still request a “control” measurement from the Lab. Indeed, large differences between POC testing and Lab have been reported in the literature, particularly for electrolytes [6–11]. Several factors may contribute to such discrepancies. First, the pre-analytical phase has a high risk of error, especially with blood gas analysis. Arterial drawing of samples should be performed under anaerobic conditions. The sample should be homogenized to prevent any clot formation and analysis should be performed without delay to prevent any metabolism occurring within the sampled cells [12]. Second, the method of measurement also matters. Both POC testing and Lab analyzers used Ion-selective electrode (ISE) technology for electrolytes assay; however, the measurement is performed on whole-blood samples (direct) for the former and on diluted plasma (indirect) for the latter [13, 14]. In patients with hypoproteinemia, a higher level of sodium has been reported with the indirect ISE assay [15–17]. In contrast, bicarbonate, glucose, and hemoglobin are usually determined using different methods. For glucose, differences seem to be related, at least in part, to hematocrit and pH level [18, 19].

Third, some methodological issues may preclude comparisons between POC testing and Lab analyzers. For example, some studies have retrospectively used a hospital’s database, whereas others have compared arterial with venous blood samples. To date, only one study has investigated the accuracy of the latest generation Siemens RAPIDPoint 500 blood gas system but this comparison was with a previous model from the same manufacturer that had similar electrochemical sensors [20]. Therefore, it seems inappropriate to draw evidence-based conclusion regarding the interchangeability of assays.

We conducted this prospective observational study in critically ill patients to address the agreement between a POC blood gas system and central Lab analyzers to measure electrolytes, bicarbonate, hemoglobin, hematocrit and glucose. We also aimed to investigate the cause of any discordance between techniques.

## Materials and Methods

The protocol of this study has been registered at https://clinicaltrials.gov/ (NCT 02449226) and recorded by the French Health Authority (ANSM, ID RCB number: 2015-A00718-41). An independent ethics committee (Comité de Protection des Personnes Sud-Méditérranée I) has approved the protocol and has waived the need for written informed consent. Oral information was provided to patients or their next of kin before enrollment.

### Patients and samples

All samples were collected prospectively over 1-month (June 2015) from patients admitted into the ICU of the Hôpital Européen Marseille. Patients were included in the study, on a daily basis, if the attending physician had ordered both biological analyses and arterial blood gases, and if an indwelling arterial catheter was present. All samples were drawn simultaneously from an arterial line in the morning (06:00 am) by nurses. BD Vacutainer tubes were collected after withdrawing 5 mL of blood and processed within two hours at the central Lab. A 3 mL BD Preset heparinized-syringe was filled and analyzed immediately in the ICU using a POC Siemens RAPIDPoint 500 blood gas system.

### Central laboratory measurements (Lab)

All analyses were performed in the Laboratoire Européen which is located within the Hospital. Biochemical parameters were determined using Beckman& Coulter AU 5800 chemistry analyzers and hematological parameters with Beckman Coulter UniCel DxH 800 automated analyzers. Electrolytes (Na^+^, K^+^, and Cl^-^) were determined using the indirect ISE method. Bicarbonate and glucose were determined using the UV-enzymatic method (phosphoenolpyruvate and hexokinase, respectively). Total proteins were determined by colorimetry (Biuret method). Hemoglobin (Hb) was determined by photometry at 525 nm after chemical lyse of red cells. Hematocrit (Hct) was calculated as the number of red cells (RBC) per the mean cell volume (MCV), such as: Hct = (RBC × MCV) / 10. The lactescence, hemolysis, and icteric indexes, automatically displayed by the chemical AU 5800 analyzers, were also collected prospectively.

### RAPIDPoint 500 measurements (POC)

Two POC RAPIDPoint 500 blood gas systems were available in the ICU. The devices work on a maintenance-free basis with 28-day single-use multiple cartridges. Of the three mounted cartridges, one is used for the measurement and contains miniaturized electro-chemical sensors, one is for washing and wasted fluids, and the third is for calibrations and internal quality control, and contains calibrated solutions. According to the size of the cartridge, the number of measurements ranged from 250 to 750. The analyzer automatically calibrates sensors several times a day, such as: a 1-point calibration every 30 min, a 2-point calibration every 2 hours, and a complete calibration every 8 hours. Analyses were performed on 200 μL of whole-blood sample and results were available in 60 s. Electrolytes were determined using the direct ISE method (potentiometry). Actual bicarbonate ion (HCO_3_^-^_act_) was calculated using the following formula: HCO_3_^-^_act_ = 0,0307 × PCO_2_ × 10^(pH– 6,105)^ where PCO_2_ is carbon dioxide tension and pH is potential hydrogen. Glucose was determined by amperometry (glucose-oxidase). Total hemoglobin was determined by multiwavelength spectrophotometry and hematocrit was estimated according to the following formula: Hct = Hb × 2.941.

### Inter-assay imprecision

Coefficients of variations (CVs) were determined daily over a 30-day period as part of routine quality control practice and are presented in Tables 1 and 2. CVs obtained at different levels were averaged and used for the Deming regression. The between-day imprecision for POC hemoglobin and POC actual bicarbonate tests cannot be measure directly because these parameters are calculated according to the above mentioned formulas. The relationship between hematocrit and hemoglobin being linear, we used the CV of hematocrit for the purpose of hemoglobin. To determine the CV of actual bicarbonate, we computed each value of HCO_3_^-^ according to the respective pH and PaCO_2_ values (see the formula above), this at each of the three control level.

**Table 1:** Between-day imprecision of the Point-of-Care Siemens RAPIDPoint 500 blood gas system (POC).

	Level 1			Level 2			Level 3			Average CV (%)
	QC	Mean	CV (%)	QC	Mean	CV (%)	QC	Mean	CV (%)	
**Na**^**+**^**, mmol/L**	115	115.2	0.32	135	135	0.21	155	155	0.3	0.28
**K**^**+**^**, mmol/L**	3	3	0.53	5	5	0.28	7	7	0.21	0.34
**Cl**^**-**^**, mmol/L**	80	80	0.23	100	99.2	0.82	120	117.8	1.25	0.77
**pH**	7.15	7.15	0.06	7.35	7.35	0.04	7.55	7.55	0.06	0.05
**PCO**_**2**_**, mmHg**	70	68.4	1.73	40	40	1.47	22	22.2	2.63	1.94
**HCO**_**3**_^**-**^**, mmol/L**	ND	ND	1.8	ND	ND	1.35	ND	ND	2.37	1.84
**Glu, mg/dL**	200	199.1	1.65	100	99.3	1.65	50	49.5	1.35	1.55
**Hb, g/dL**	18	18	0.31	14	14	0.38	8	8	0.42	0.34
**Hct, %**	ND	ND	0.31	ND	ND	0.38	ND	ND	0.42	0.34

ND is Not Done, QC is quality control (target), CV is Coefficient of Variation, Na^+^ is sodium, K^+^ is potassium, Cl^-^ is chloride, PCO_2_ is partial pressure of CO_2_, HCO_3_^-^ is bicarbonate, Glu is glucose, Hb is hemoglobin, and Hct is hematocrit.

**Table 2:** Between-day imprecision of central laboratory automated analyzers (Lab).

	Level 1			Level 2			Level 3			Average CV (%)
	QC	Mean	CV (%)	QC	Mean	CV (%)	QC	Mean	CV (%)	
**Na**^**+**^**, mmol/L**	122	122.8	0.7	ND	ND	ND	153	153.4	0.7	0.7
**K**^**+**^**, mmol/L**	3.93	3.96	0.7	ND	ND	ND	6.64	6.59	1.1	0.85
**Cl**^**-**^**, mmol/L**	89.9	90	0.65	ND	ND	ND	111	110.8	0.75	0.7
**HCO**_**3**_^**-**^**, mmol/L**	10	10.7	3.5	16	17.4	3.9	27	30	2.2	3.2
**Glu, mg/dL**	103	101	1.65	ND	ND	ND	240	240	1.35	1.5
**Hb, g/dL**	4.6	4.7	1.13	12	11.9	1.02	15.6	15.5	1.01	1.05
**Hct, %**	14.4	14.8	0.9	36.1	36	0.86	47.6	47.8	0.9	0.89

ND is Not Done, QC is quality control (target), CV is Coefficient of Variation, Na^+^ is sodium, K^+^ is potassium, Cl^-^ is chloride, HCO_3_^-^ is bicarbonate, Glu is glucose, Hb is hemoglobin, and Hct is hematocrit.

### Statistical methods

Data are expressed as mean ± standard deviation (SD) unless specified. Statistical analyses were conducted according to the NCCLS-EP9-A3 guideline [21]. We decided to not exclude outliers. Agreement between Lab and POC measurements was investigated using the Bland-Altman method with multiple observations per individual, including bias and limit of agreement (± 1.96 SD) [22]. We performed Deming regression analysis, which assumes random errors in both measurement procedures [23]. Precision was determined by Pearson’s coefficient of correlation (r) and determination (r^2^), and accuracy by the bias correction factor (Cb) and the concordance correlation coefficient (κ). We used the proficiency testing (PT) criteria to define acceptable analytical performance (CLIA, 1992) with the following cut-off values [24]: ± 4 mmol/L for Na^+^; ± 0.5 mmol/L for K^+^; ± 5% for Cl^-^; ±6 mg/dL or ± 10% for glucose; ± 6% for hematocrit; and ± 7% for hemoglobin. No cut-off value was available for HCO_3_^-^. As suggested by CLIA, differences between means were expressed either in units or as percentages.

To further investigate the cause for any differences between the techniques, we used linear regression analyses to quantify the relationship with protein, hematocrit, and pH. Statistical analyses were performed using MedCalc for Windows v15.8 (MedCalc Software, Ostend, Belgium). A *p* value of <0.05 was considered statistically significant.

## Results

We collected 314 paired samples from 51 critically ill patients. The mean age was 69±12 years, and 32 (69%) were male. The mean SAPS II score (Simplified Acute Physiology Score, ranging from 0 to 150, with higher value indicating greater severity) was 47±21. Twenty-four patients (47%) received vasopressor, 34 (67%) received mechanical ventilation, and 14 (27%) did not survive the ICU. Among the 314 collected paired samples, one was not complete for chloride analysis, one not for bicarbonate, eight not for glucose, and two not for hemoglobin and hematocrit (analyses not ordered at Lab). The lactescence index was positive in one sample, the hemolysis index positive in 7, and the icteric index positive in 48 (whatever the values on a 0 to 10-point scale). The maximum value of the lactescence index was 1; the maximum of the hemolysis index was 2, and the maximum of the icteric index was 4. There was no interfering substance during POC analyses. The Dataset of the present study is available in a S1 Dataset accompanying the manuscript. The range, mean, median and SD of variables of interest are presented in Table 3. The coefficient of precision, determination, accuracy, and concordance are presented in Table 4.

**Table 3:** Range, mean, median and standard deviation according to assays (POC and Lab).

	Range (min–max)		Mean		Median		SD	
	POC	Lab	POC	Lab	POC	Lab	POC	Lab
**Na**^**+**^**, mmol/L**	119.4–150.2	121–152	135.4	136.7	134.7	136	5	4.7
**K**^**+**^**, mmol/L**	2.8–5.8	2.9–5.9	3.9	4.1	3.8	4.1	0.6	0.6
**Cl**^**-**^**, mmol/L**	86–116	86–115	102.1	101.2	102	101	5.6	5.6
**HCO**_**3**_^**-**^**, mmol/L**	13.5–43.1	14–43	25.2	26	24.2	25	5.3	5.4
**Glu, mg/dL**	50–397	32–373	135.4	124.8	129	119	41.5	41
**Hb, g/dL**	6.1–15.5	5.6–14.7	10.3	9.5	10.2	9.4	1.6	1.5
**Hct, %**	18–46	16.9–45.2	30.2	29.1	30	28.8	4.7	4.8

POC is Point-of-Care Siemens RAPIDPoint 500 blood gas system, Lab is central Laboratory analyzers, SD is standard deviation, Na^+^ is sodium (n = 314), K^+^ is potassium (n = 314), Cl^-^ is chloride (n = 313), HCO_3_^-^ is bicarbonate (n = 313), Glu is glucose (n = 306), Hb is hemoglobin (n = 312) and Hct is hematocrit (n = 312).

**Table 4:** Coefficient of precision, determination, concordance and accuracy between assays (POC and Lab).

	Precision	Determination	Concordance	Accuracy
	r	r^2^	κ	C_b_
**Na**^**+**^**, mmol/L**	0.9359	0.8759	0.9013	0.9630
**K**^**+**^**, mmol/L**	0.9443	0.8917	0.8888	0.9412
**Cl**^**-**^**, mmol/L**	0.9669	0.9348	0.9553	0.988
**HCO**_**3**_^**-**^**, mmol/L**	0.9678	0.9366	0.9578	0.9896
**Glu, mg/dL**	0.9716	0.944	0.9399	0.9673
**Hb, g/dL**	0.9851	0.9704	0.8728	0.8861
**Hct, %**	0.9814	0.9631	0.9561	0.9743

POC is Point-of-Care Siemens RAPIDPoint 500 blood gas system, Lab is central Laboratory analyzers, Na^+^ is sodium (n = 314), K^+^ is potassium (n = 314), Cl^-^ is chloride (n = 313), HCO_3_^-^ is bicarbonate (n = 313), Glu is glucose (n = 306), Hb is hemoglobin (n = 312) and Hct is hematocrit (n = 312).

### Sodium

**Fig 1:**
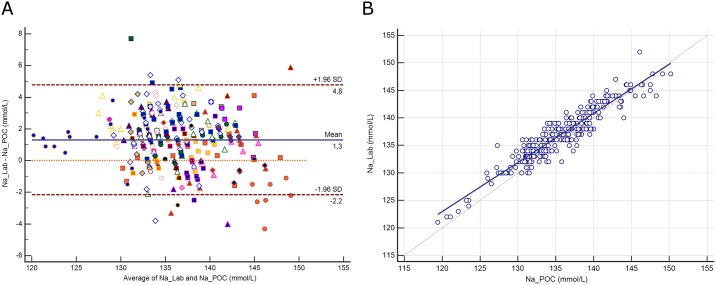
**1A.** Bland-Altman plot showing the difference versus the average of sodium (Na) measured by the central Lab analyzer and by the POC Siemens RAPIDPoint 500 blood gas system. Bias, upper and lower limits of agreement (±1.96 SD) are represented. The red dotted line indicates equality (difference = 0). There is one marker for each patient. **1B**. Scatter diagram showing Deming regression analysis for sodium (Na) measured by the central Lab analyzer and by the POC Siemens RAPIDPoint 500 blood gas system. The regression line (solid line) and the identity line (x = y, dotted line) are represented.

The distribution of sodium (Na) and its difference between assays are presented in S1A and S1B Fig. The mean difference between assays was 1.3 mmol/L (±1.96 SD, -2.2 to 4.8 mmol/L; Fig 1A). Deming regression analysis yielded the equation: Na^+^ Lab (mmol/L) = 16 + 0.89 Na^+^ POC (Fig 1B). The difference between methods was correlated with protein (r^2^ = 0.48, p<0.0001) and hematocrit (r^2^ = 0.1, *p*<0.0001). A difference above the CLIA criteria was noted for 18 paired samples (5.8%).

### Potassium

**Fig 2:**
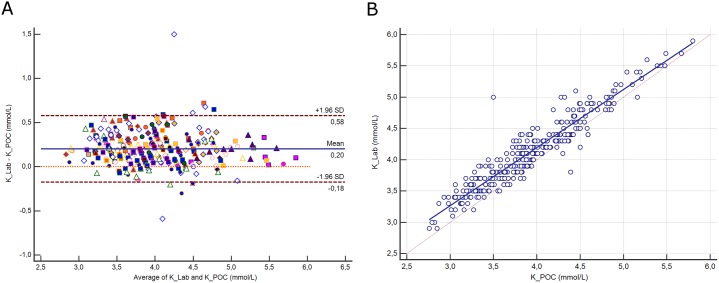
**2A.** Bland-Altman plot showing the difference versus the average of potassium (K) measured by the central Lab analyzer and by the POC Siemens RAPIDPoint 500 blood gas system. Bias, upper and lower limits of agreement (±1.96 SD) are represented. The red dotted line indicates equality (difference = 0). There is one marker for each patient. **2B**. Scatter diagram showing Deming regression analysis for sodium potassium (K) measured by the central Lab analyzer and by the POC Siemens RAPIDPoint 500 blood gas system. The regression line (solid line) and the identity line (x = y, dotted line) are represented.

The distribution of potassium (K) and its difference between assays are presented in S2A and S2B Fig. The mean difference between assays was 0.2 mmol/L (±1.96 SD, -0.18 to 0.58 mmol/L; Fig 2A). Deming regression analysis yielded the equation: K^+^ Lab (mmol/L) = 0.474 + 0.929 K^+^ POC (Fig 2B). No correlation with protein was observed (*p* = 0.42). A difference above the CLIA criteria was noted for 18 paired samples (5.8%).

### Chloride

**Fig 3:**
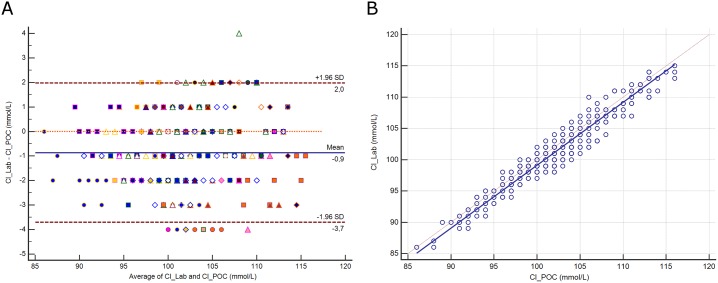
**3A.** Bland-Altman plot showing the difference versus the average of chloride (Cl) measured by the central Lab analyzer and by the POC Siemens RAPIDPoint 500 blood gas system. Bias, upper and lower limits of agreement (±1.96 SD) are represented. The red dotted line indicates equality (difference = 0). There is one marker for each patient. **3B**. Scatter diagram showing Deming regression analysis for chloride (Cl) measured by the central Lab analyzer and by the POC Siemens RAPIDPoint 500 blood gas system. The regression line (solid line) and the identity line (x = y, dotted line) are represented.

The distribution of chloride (Cl) and its difference between assays are presented in S3A and S3B Fig. The mean difference between assays was -0.9 mmol/L (±1.96 SD, -3.7 to 2 mmol/L; Fig 3A), representing a relative difference of 0.9%. Deming regression analysis yielded the equation: Cl^-^ Lab (mmol/L) = -1.329 + 1.005 Cl^-^ POC (Fig 3B). The difference between methods was correlated with protein (r^2^ = 0.11, *p*<0.0001).

### Bicarbonate

**Fig 4:**
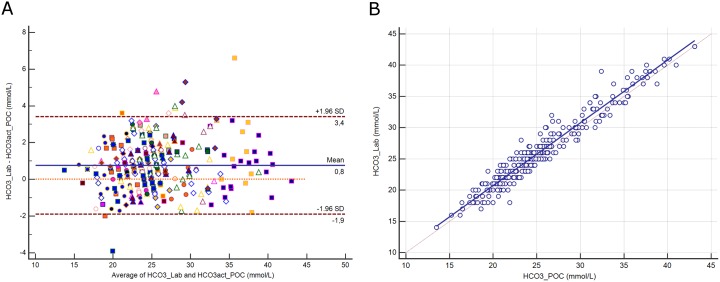
**4A.** Bland-Altman plot showing the difference versus the average of bicarbonate (HCO_3_) measured by the central Lab analyzer and by the POC Siemens RAPIDPoint 500 blood gas system. Bias, upper and lower limits of agreement (±1.96 SD) are represented. The red dotted line indicates equality (difference = 0). There is one marker for each patient. **4B**. Scatter diagram showing Deming regression analysis for bicarbonate (HCO_3_) measured by the central Lab analyzer and by the POC Siemens RAPIDPoint 500 blood gas system. The regression line (solid line) and the identity line (x = y, dotted line) are represented.

The distribution of bicarbonate (HCO_3_) and its difference between assays are presented in S4A and S4B Fig. The mean difference between assays was 0.8 mmol/L (±1.96 SD, -1.9 to 3.4 mmol/L; Fig 4A). Deming regression analysis yielded the equation: HCO_3_^-^ Lab (mmol/L) = 0.642 + 1.005 HCO_3_^-^ POC (Fig 4B).

### Glucose

**Fig 5:**
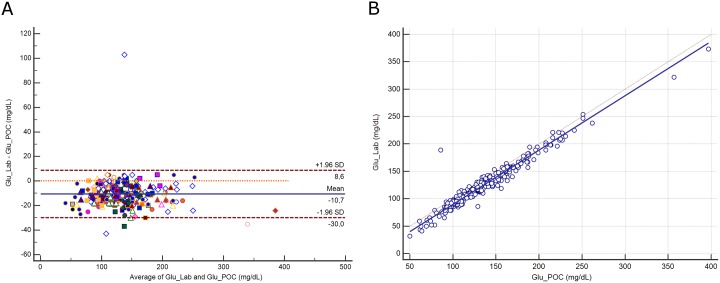
**5A.** Bland-Altman plot showing the difference versus the average of glucose (Glu) measured by the central Lab analyzer and by the POC Siemens RAPIDPoint 500 blood gas system. Bias, upper and lower limits of agreement (±1.96 SD) are represented. The red dotted line indicates equality (difference = 0). There is one marker for each patient. **5B**. Scatter diagram showing Deming regression analysis for glucose (Glu) measured by the central Lab analyzer and by the POC Siemens RAPIDPoint 500 blood gas system. The regression line (solid line) and the identity line (x = y, dotted line) are represented.

The distribution of glucose (Glu) and its difference between assays are presented in S5A and S5B Fig. The mean difference between assays was -10.7 mg/dL (±1.96 SD, -30 to 8.6 mg/dL; Fig 5A), representing a relative difference of 8%. Deming regression analysis yielded the equation: Glu Lab (mg/dL) = -9.64 + 0.992 Glu POC (Fig 5B). The difference between methods was correlated with hematocrit (r^2^ = 0.07, *p*<0.0001) but not with pH (*p* = 0.8). A difference above the CLIA criteria was noted for 129 paired samples (42.3%).

### Hemoglobin

**Fig 6:**
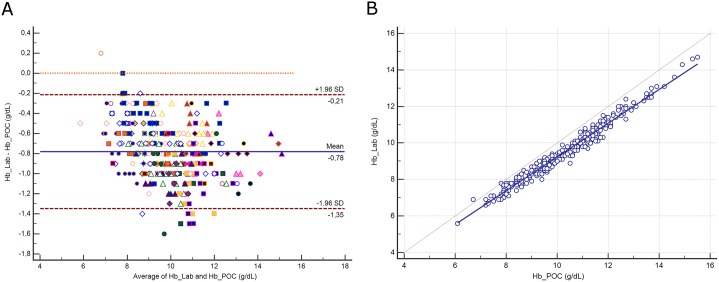
**6A.** Bland-Altman plot showing the difference versus the average of hemoglobin (Hb) measured by the central Lab analyzer and by the POC Siemens RAPIDPoint 500 blood gas system. Bias, upper and lower limits of agreement (±1.96 SD) are represented. The red dotted line indicates equality (difference = 0). There is one marker for each patient. **6B**. Scatter diagram showing Deming regression analysis for hemoglobin (Hb) measured by the central Lab analyzer and by the POC Siemens RAPIDPoint 500 blood gas system. The regression line (solid line) and the identity line (x = y, dotted line) are represented.

The distribution of hemoglobin (Hb) and its difference between assays are presented in S6A and S6B Fig. The mean difference between assays was -0.78 g/dL (±1.96 SD, -1.35 to -0.21 g/dL; Fig 6A), representing a relative difference of 8.2%. Deming regression analysis yielded the equation: Hb Lab (g/dL) = -0.02 + 0.926 Hb POC (Fig 6B). The difference between methods was correlated with protein (r^2^ = 0.19, *p*<0.0001) and hematocrit (r^2^ = 0.14, *p*<0.0001). A difference above the CLIA criteria was noted for 203 paired samples (65%).

### Hematocrit

**Fig 7:**
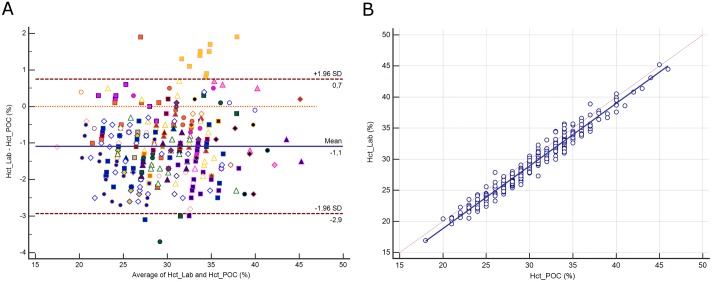
**7A.** Bland-Altman plot showing the difference versus the average of hematocrit (Hct) measured by the central Lab analyzer and by the POC Siemens RAPIDPoint 500 blood gas system. Bias, upper and lower limits of agreement (±1.96 SD) are represented. The red dotted line indicates equality (difference = 0). There is one marker for each patient. **7B**. Scatter diagram showing Deming regression analysis for hematocrit (Hct) measured by the central Lab analyzer and by the POC Siemens RAPIDPoint 500 blood gas system. The regression line (solid line) and the identity line (x = y, dotted line) are represented.

The distribution of hematocrit (Hct) and its difference between assays are presented in S7A and S7B Fig. The mean difference between assays was -1.1% (±1.96 SD, -2.9 to 0.7%; Fig 7A), representing a relative difference of 3.7%. Deming regression analysis yielded the equation: Hct Lab (%) = -1.353 + 1.008 Hct POC (Fig 7B). A difference above the CLIA criteria was noted for 86 paired samples (27.6%).

## Discussion

In the present study, we have demonstrated that POC Siemens RAPIDPoint 500 systems and central Laboratory analyzers fulfilled CLIA PT criteria’s for all tested parameters, except for hemoglobin. However, these results are warranted only for a careful pre-analytical management of samples.

More or less large differences have been reported in studies that have compared direct and indirect ISE assays to measure electrolytes, especially for sodium [6–11]. The indirect ISE assay consists of a fixed predilution step with a buffer solution. Normally, serum contains a water phase (93%) and a non-water or solid phase (7% by volume); the latter being mainly constituted by proteins and lipids. During the dilution step, the same volume of diluent is always used and the degree of dilution is estimated on the basis of the expected solid fraction (7%). However, if the solid fraction decreases, as during hypoproteinemia, dilution becomes less and measurement of an ion is then overestimated [14, 25]. Hypoproteinemia, as defined by a total protein content of <60 g/L, was present in 240 (76%) of our samples. We observed, as others have, higher sodium levels with the indirect ISE assay (mean 1.3 mmol/L) whereas change in the level of protein accounted for 48% of the difference between assays [15–17]. Eighteen (5.8%) of our sodium paired samples were found to be outside the CLIA cut-off value (± 4 mmol/L). Using the international classification for hyponatremia [25], errors in staging would have occurred in two patients with severe hyponatremia, 15 with moderate hyponatremia, and 55 with mild hyponatremia. In contrast, there was no discordance in the diagnosis of hypernatremia (>145 mmol/L).

Potassium and chloride measurements were less affected by the presence of hypoproteinemia because we found a weak correlation for chloride and none for potassium. However, among the 18 (5.8%) potassium paired samples that were outside the CLIA cut-off value of ±0.5 mmol/L, hypoproteinemia was present in 17. The normal range for plasma potassium level is 3.5–5 mmol/L [26]. In our study, a potassium level of < 3.5 mmol/l was observed in 85 samples when measured by the direct ISE assay (POC) and in only 33 samples using indirect ISE (Lab). Severe hypokalemia (<3 mmol/L) was observed in eight samples when measured by POC and in only two samples when measured by Lab. Conversely, a potassium level of >5 mmol/L was observed in 13 samples measured by POC and in 17 measured by Lab. Finally, severe hyperkalemia (>5.5 mmol/L) was present in two samples when measured by POC and in four samples measured by Lab. Differences in chloride had never exceeded the CLIA cut-off limit of ±5%. For all electrolytes, the mean difference between assays remained below the respective CLIA criteria, defining interchangeability. Because the direct ISE assay is not influenced by protein content, we wonder whether POC blood gas analyzes should be the reference, provided that errors in the pre-analytical phase are minimized [17].

In the present study, we also compared the calculated (actual) and the measured (serum) bicarbonate concentrations, with the latter being the reference method. Of note, the total carbon dioxide content (including bicarbonate) is higher in venous than in arterial blood [27]. Kumar and co-workers reported, in a retrospective analysis of 17621 samples, a mean difference of -0.36 mmol/L between measured (venous blood) and calculated (arterial blood) bicarbonate [28]. They found that 95.3% of the paired samples had a difference of ≤2 mmol/L and only 0.65% of samples were considered as being clinically discordant (≥4 mmol/L). We were the first to compare arterial blood specimens and reported a mean difference of 0.8 mmol/L. We observed a difference of ≥2 mmol/L in 56 samples (18%) whereas it exceeded 4 mmol/L in only five samples (1.6%). Although a CLIA cut-off has not been established for the purpose of bicarbonate, we may consider that clinical discordance between the two methods is uncommon, which plea for their interchangeability.

Glycemic control is part of routine care for critically ill patients because hyperglycemia is frequent and is associated with a worse outcome [29]. International guidelines recommend a target serum glucose level of between 80 and 180 mg/dL [30]. Insulin is the first-line therapy to control hyperglycemia but is associated with a risk of hypoglycemia [31]. Although the reference method (hexokinase) is used only by central Lab analyzers, the POC blood gas amperometric method (glucose oxidase) appears to be the most reliable alternative [32, 33]. Using the CLIA criterion of ± 6 mg/dL for interchangeability, the difference between methods was higher than the cut-off value in 229 (75%) of the 306 paired samples. However, when using the alternative CLIA criterion of ±10%, only 129 (42%) samples had a difference higher than the cut-off value. The whole difference between samples (8%) remained below the limit for interchangeability only if defined as ±10%. Moderate hypoglycemia (<60 mg/dL) occurred in one sample measured by POC and in nine measured by Lab. Only one sample was within the severe hypoglycemia zone (<40 mg/dL) when measured by Lab and none by POC. The mean difference between these methods was 10.7 mg/dL, which is in line with data from Pereira et al, who found a difference of 14 mg/dL [18]. These authors suggested that hematocrit and pH may interfere with the amperometric method. We found only a relation with hematocrit, which accounted for 7% of the difference between the methods.

We also investigated the concordance of the hemoglobin assays. The POC RAPIDPoint 500 analyzer quantified the total hemoglobin as the sum of the four moieties (HbO_2_, HbH, MetHb and HbCO) using spectrophotometric analysis of whole-blood. The DxH 800 analyzer measured hemoglobin spectrophotometrically as the absorbance at 525 nm of the colored HemoChrom-S complex. Although the reference method for hemoglobinometry is spectrophotometry using hemiglobin-cyanide, cyanide-free reagents have been widely adopted for safety and environmental purpose [34, 35]. Therefore, the DxH 800 analyzer represents current standards [36, 37]. We found that hemoglobin was systematically overestimated (mean 0.8 g/dL) by the POC assay. This result is consistent with the study of Frasca et al who observed a mean difference of 0.9 g/dL using a previous model (RAPIDPoint 405) [38]. Among potential confounders, high lipid fractions, cell fragments from incomplete hemolysis and high bilirubin level are recognized [39]. In our population, the difference between hemoglobin assays was not related to any of those (i.e., the lactescence, hemolysis and icteric index) but to protein and hematocrit, which account together for 26% of the difference. Therefore, we assumed that the POC method was the main confounder. Transfusion threshold has moved toward lower values in critically ill patients (hemoglobin ≤ 7 g/dL) since the publication of the TRISS trial [40]. In our population, ten (3%) samples were ≤7 g/dL when measured by Lab and only two when measured by POC. From a clinical point of view, a transfusion of packed red blood cells wouldn't have been infused while needed in eight cases, if any physicians had relied on the POC hemoglobin assay. Hemoglobin was the only parameter to not fulfill the CLIA criteria of interchangeability with a mean difference between methods of 8.2% when the cut-off was 7%. Among our 312 paired samples, 203 (65%) were above the CLIA cut-off value.

Finally, we compared the hematocrit assays. The DxH 800 analyzer applied the Coulter principle to determine the red cell counts and the mean cellular volume. The RAPIDPoint 500 analyzer assumed a constant mean corpuscular hemoglobin concentration (2.941) and estimated hematocrit in proportion to the hemoglobin. If the transfusion threshold is based on the hematocrit, as in cardiac surgery [39], a hematocrit of ≤21% was observed in nine (3%) samples measured by Lab and in 6 measured by POC. Among our 312 paired samples, 86 (28%) were above the CLIA cut-off value of 6%. Nevertheless, the mean difference between methods remained below the CLIA criteria.

### Limitations and strengths

Although we attempted to minimize inadequate pre-analytical management by delivering a 1-month preliminary teaching program, we did not assess compliance of health-care workers. Pre-analytical errors may have contributed to outliers. We used the CLIA PT criteria which were defined back in the 1980s and reflected the state of the art at that time. Even if inappropriate nowadays, they have still not been updated. Last, our results should be restricted to studied devices.

Our study also has several strengths. We collected all samples prospectively, through an arterial line, and simultaneously for both analyses. We avoided contamination by withdrawing 5 ml of blood prior to collect samples, a volume which is higher than three-times the dead space volume of the apparatus [41]. Analyses were performed without delay. Our samples were drawn from a representative population of critically ill patients and covered a wide range of values.

### Conclusions

In the present study, we have demonstrated that in a population of critically ill patients, POC Siemens RAPIDPoint 500 blood gas systems and central laboratory analyzers are interchangeable for the measurement of electrolytes, bicarbonate, glucose and hematocrit, but not for hemoglobin. We found a systematic overestimation of hemoglobin with the POC device suggesting an inaccuracy in the method of measurement. Although glucose values were close enough on average, we observed clinically relevant discrepancies in the range of hypoglycemia. These results are warranted for our own procedures and devices, and should be interpreted with caution in others institutions. Bearing these restrictions, we recommend clinicians to initiate an appropriate therapy based on POC testing without awaiting a control measurement.

## Supporting Information

S1 Dataset DataSet of the 314 paired samples. (XLSX)

S1 Fig **S1A.** Dot plot showing the distribution of sodium (Na) measured by the central Lab analyzer (Beckman& Coulter AU 5800) and by the Point-of-Care (POC) Siemens RAPIDPoint 500 blood gas system (n = 314). The solid line indicates the mean. **S1B.** Histogram showing the relative distribution of the difference between sodium (Na) measured by the central Lab analyzer and by the POC Siemens RAPIDPoint 500 blood gas system.
(TIF)

S2 Fig **S2A.** Dot plot showing the distribution of potassium (K) measured by the central Lab analyzer (Beckman& Coulter AU 5800) and by the Point-of-Care (POC) Siemens RAPIDPoint 500 blood gas system (n = 314). The solid line indicates the mean. **S2B.** Histogram showing the relative distribution of the difference between potassium (K) measured by the central Lab analyzer and by the POC Siemens RAPIDPoint 500 blood gas system.
(TIF)

S3 Fig **S3A.** Dot plot showing the distribution of chloride (Cl) measured by the central Lab analyzer (Beckman& Coulter AU 5800) and by the Point-of-Care (POC) Siemens RAPIDPoint 500 blood gas system (n = 313). The solid line indicates the mean. **S3B.** Histogram showing the relative distribution of the difference between chloride (Cl) measured by the central Lab analyzer and by the POC Siemens RAPIDPoint 500 blood gas system.
(TIF)

S4 Fig **S4A.** Dot plot showing the distribution of bicarbonate (HCO_3_) measured by the central Lab analyzer (Beckman& Coulter AU 5800) and by the Point-of-Care (POC) Siemens RAPIDPoint 500 blood gas system (n = 313). The solid line indicates the mean. **S4B.** Histogram showing the relative distribution of the difference between bicarbonate (HCO_3_) measured by the central Lab analyzer and by the POC Siemens RAPIDPoint 500 blood gas system.
(TIF)

S5 Fig **S5A.** Dot plot showing the distribution of glucose (Glu) measured by the central Lab analyzer (Beckman& Coulter AU 5800) and by the Point-of-Care (POC) Siemens RAPIDPoint 500 blood gas system (n = 306). The solid line indicates the mean. **S5B.** Histogram showing the relative distribution of the difference between glucose (Glu) measured by the central Lab analyzer and by the POC Siemens RAPIDPoint 500 blood gas system.
(TIF)

S6 Fig **S6A.** Dot plot showing the distribution of hemoglobin (Hb) measured by the central Lab analyzer (Beckman& Coulter Unicel DxH 800) and by the Point-of-Care (POC) Siemens RAPIDPoint 500 blood gas system (n = 312). The solid line indicates the mean. **S6B.** Histogram showing the relative distribution of the difference between hemoglobin (Hb) measured by the central Lab analyzer and by the POC Siemens RAPIDPoint 500 blood gas system.
(TIF)

S7 Fig **S7A.** Dot plot showing the distribution of hematocrit (Hct) measured by the central Lab analyzer (Beckman& Coulter Unicel DxH 800) and by the Point-of-Care (POC) Siemens RAPIDPoint 500 blood gas system (n = 312). The solid line indicates the mean. **S7B.** Histogram showing the relative distribution of the difference between hematocrit (Hct) measured by the central Lab analyzer and by the POC Siemens RAPIDPoint 500 blood gas system.
(TIF)
