# Supplementary figures and images for: Point-of-Care Versus Central Laboratory Measurements of Hemoglobin, Hematocrit, Glucose, Bicarbonate and Electrolytes: A Prospective Observational Study in Critically Ill Patients
Source: PLoS One. 2017 Jan 10;12(1):e0169593. doi: 10.1371/journal.pone.0169593 (PMC5224825; doi:10.1371/journal.pone.0169593)

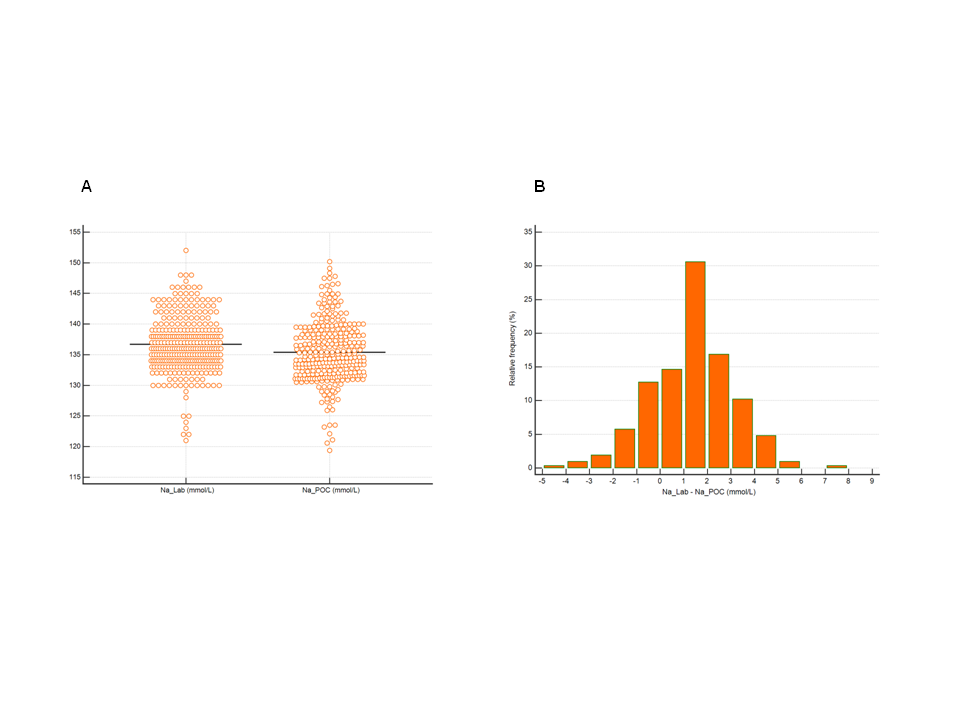

Supplement: S1 Fig — S1A. Dot plot showing the distribution of sodium (Na) measured by the central Lab analyzer (Beckman& Coulter AU 5800) and by the Point-of-Care (POC) Siemens RAPIDPoint 500 blood gas system (n = 314). The solid line indicates the mean. S1B. Histogram showing the relative distribution of the difference between sodium (Na) measured by the central Lab analyzer and by the POC Siemens RAPIDPoint 500 blood gas system. (TIF) [file pone.0169593.s002.tif]

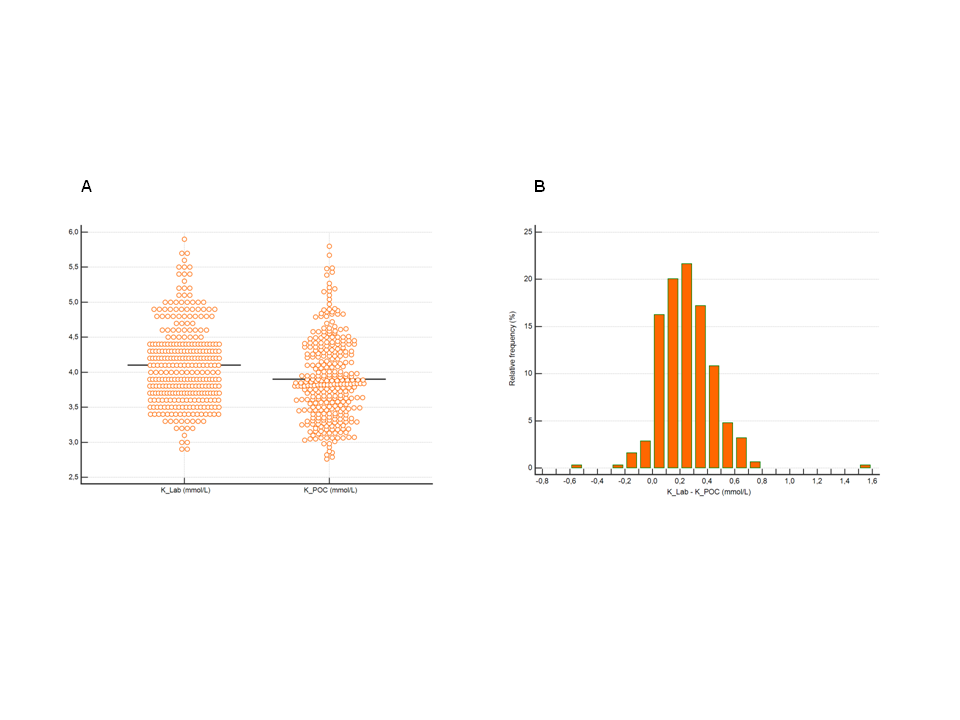

Supplement: S2 Fig — S2A. Dot plot showing the distribution of potassium (K) measured by the central Lab analyzer (Beckman& Coulter AU 5800) and by the Point-of-Care (POC) Siemens RAPIDPoint 500 blood gas system (n = 314). The solid line indicates the mean. S2B. Histogram showing the relative distribution of the difference between potassium (K) measured by the central Lab analyzer and by the POC Siemens RAPIDPoint 500 blood gas system. (TIF) [file pone.0169593.s003.tif]

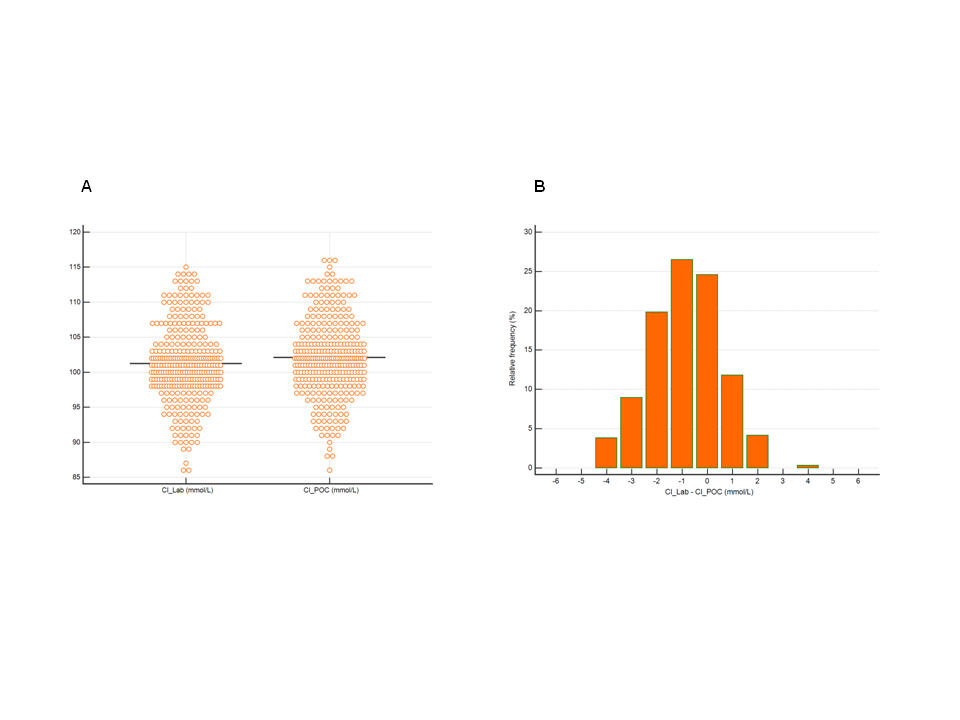

Supplement: S3 Fig — S3A. Dot plot showing the distribution of chloride (Cl) measured by the central Lab analyzer (Beckman& Coulter AU 5800) and by the Point-of-Care (POC) Siemens RAPIDPoint 500 blood gas system (n = 313). The solid line indicates the mean. S3B. Histogram showing the relative distribution of the difference between chloride (Cl) measured by the central Lab analyzer and by the POC Siemens RAPIDPoint 500 blood gas system. (TIF) [file pone.0169593.s004.tif]

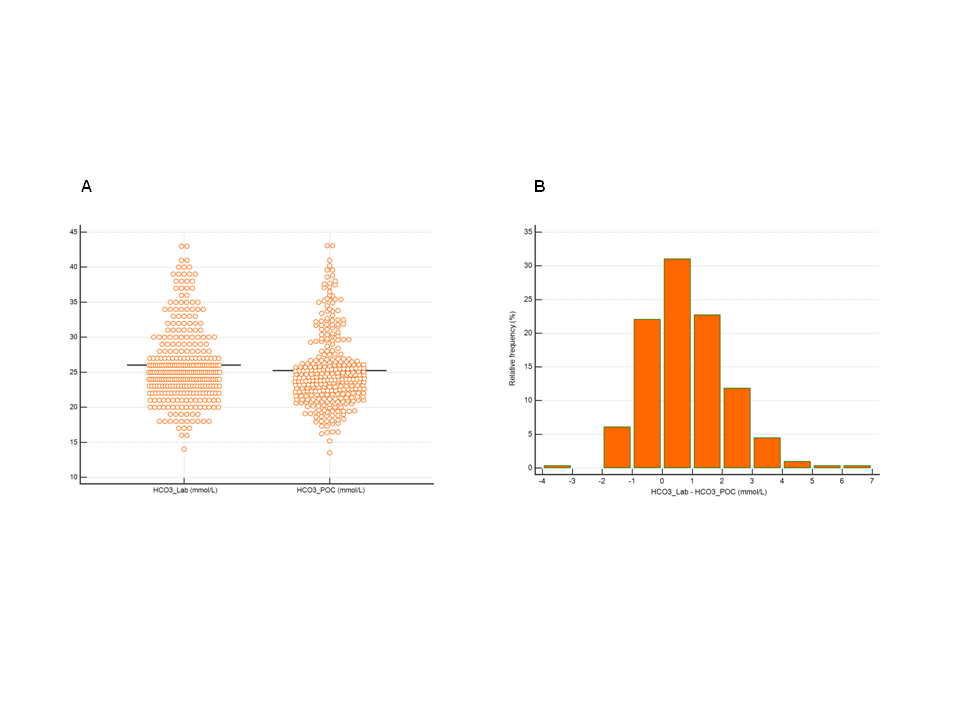

Supplement: S4 Fig — S4A. Dot plot showing the distribution of bicarbonate (HCO3) measured by the central Lab analyzer (Beckman& Coulter AU 5800) and by the Point-of-Care (POC) Siemens RAPIDPoint 500 blood gas system (n = 313). The solid line indicates the mean. S4B. Histogram showing the relative distribution of the difference between bicarbonate (HCO3) measured by the central Lab analyzer and by the POC Siemens RAPIDPoint 500 blood gas system. (TIF) [file pone.0169593.s005.tif]

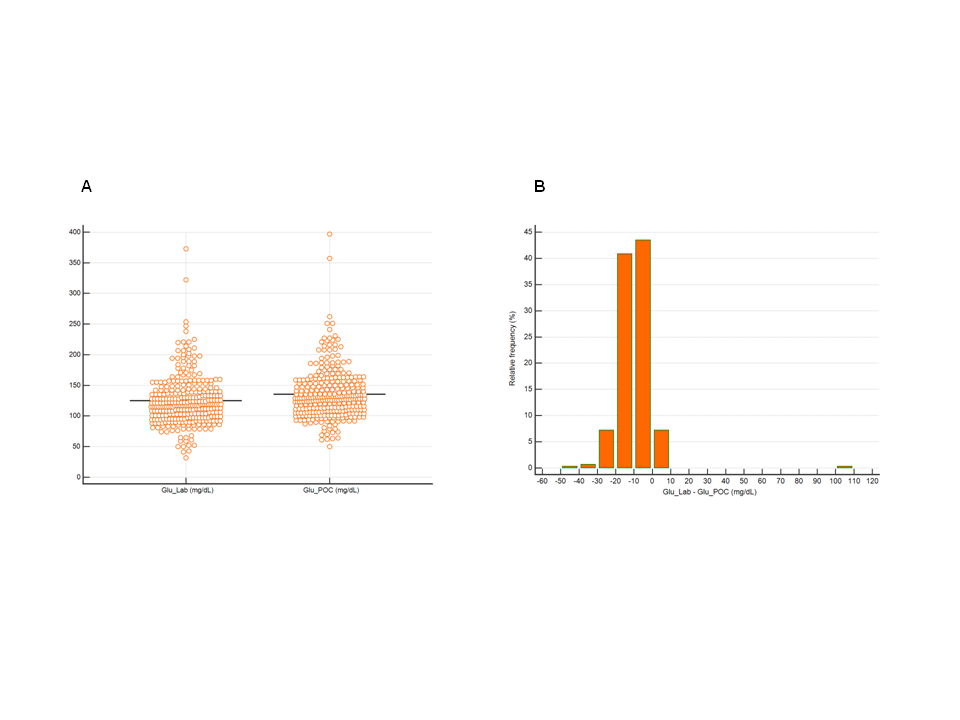

Supplement: S5 Fig — S5A. Dot plot showing the distribution of glucose (Glu) measured by the central Lab analyzer (Beckman& Coulter AU 5800) and by the Point-of-Care (POC) Siemens RAPIDPoint 500 blood gas system (n = 306). The solid line indicates the mean. S5B. Histogram showing the relative distribution of the difference between glucose (Glu) measured by the central Lab analyzer and by the POC Siemens RAPIDPoint 500 blood gas system. (TIF) [file pone.0169593.s006.tif]

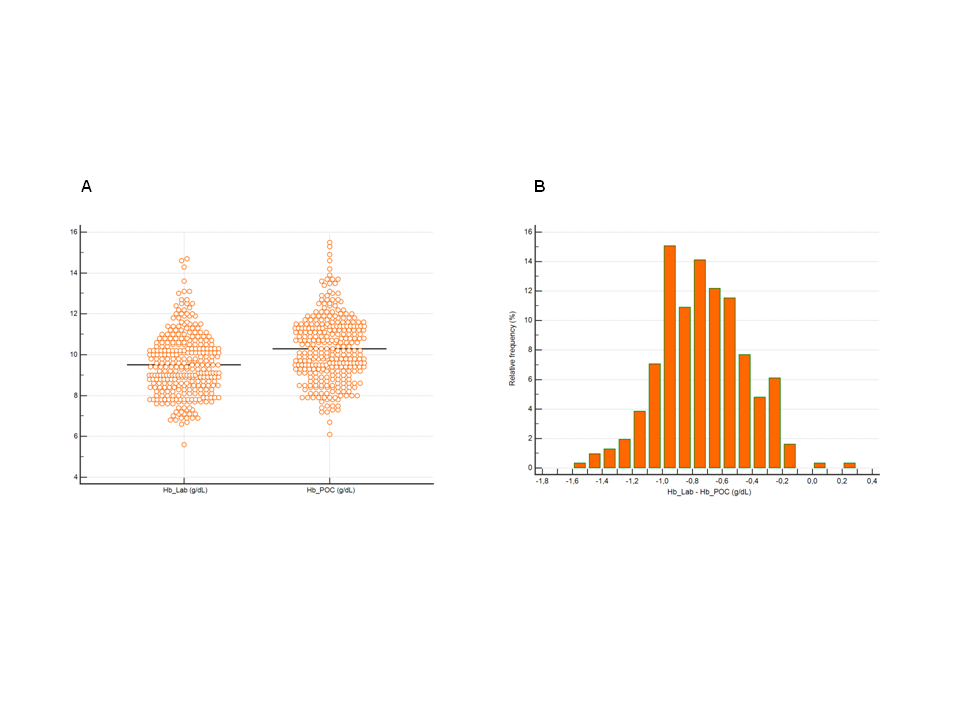

Supplement: S6 Fig — S6A. Dot plot showing the distribution of hemoglobin (Hb) measured by the central Lab analyzer (Beckman& Coulter Unicel DxH 800) and by the Point-of-Care (POC) Siemens RAPIDPoint 500 blood gas system (n = 312). The solid line indicates the mean. S6B. Histogram showing the relative distribution of the difference between hemoglobin (Hb) measured by the central Lab analyzer and by the POC Siemens RAPIDPoint 500 blood gas system. (TIF) [file pone.0169593.s007.tif]

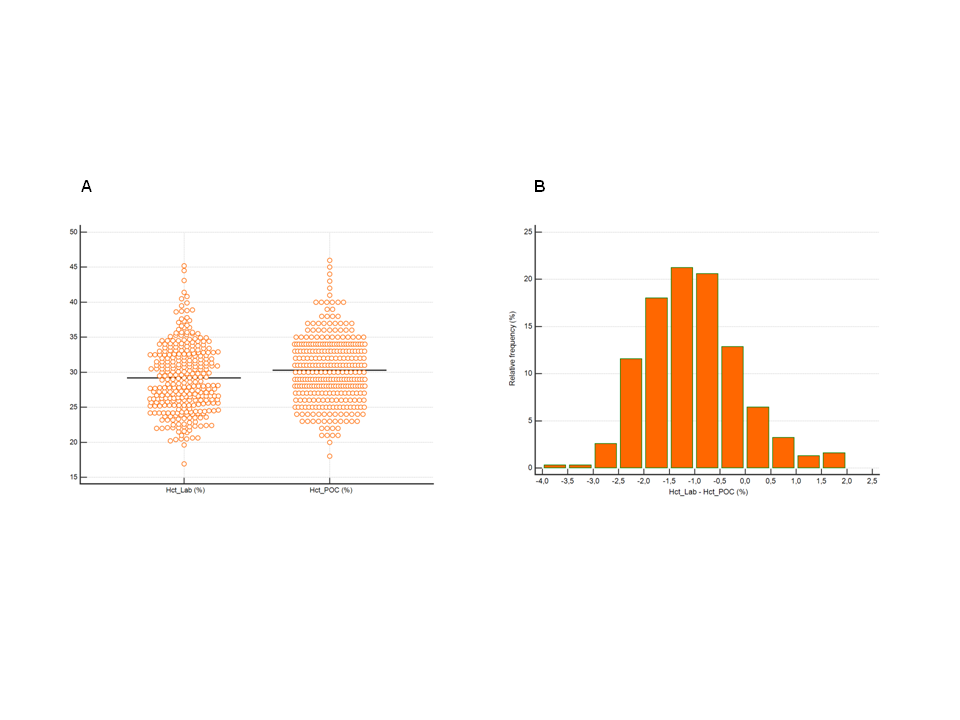

Supplement: S7 Fig — S7A. Dot plot showing the distribution of hematocrit (Hct) measured by the central Lab analyzer (Beckman& Coulter Unicel DxH 800) and by the Point-of-Care (POC) Siemens RAPIDPoint 500 blood gas system (n = 312). The solid line indicates the mean. S7B. Histogram showing the relative distribution of the difference between hematocrit (Hct) measured by the central Lab analyzer and by the POC Siemens RAPIDPoint 500 blood gas system. (TIF) [file pone.0169593.s008.tif]
